# Diagnosis of Alzheimer’s disease with Electroencephalography in a differential framework

**DOI:** 10.1371/journal.pone.0193607

**Published:** 2018-03-20

**Authors:** Nesma Houmani, François Vialatte, Esteve Gallego-Jutglà, Gérard Dreyfus, Vi-Huong Nguyen-Michel, Jean Mariani, Kiyoka Kinugawa

**Affiliations:** 1 SAMOVAR, Télécom SudParis, CNRS, Université Paris-Saclay, 9 rue Charles Fourier EVRY, France; 2 UMR CNRS 8249 Brain Plasticity Laboratory, Paris, France; 3 ESPCI Paris, PSL Research University, Paris, France; 4 Data and Signal Processing Research Group, U Science Tech, University of Vic–Central University of Catalonia, Vic, Catalonia, Spain; 5 AP-HP, DHU FAST, GH Pitié-Salpêtrière-Charles Foix, Functional Exploration Unit of older patients, Ivry-sur-Seine, France; 6 Sorbonne Universités, UPMC Univ Paris 06, UMR 8256, Biological Adaptation and Ageing, Paris, France; 7 CNRS, UMR 8256, Biological Adaptation and Ageing, Paris, France; Nathan S Kline Institute, UNITED STATES

## Abstract

This study addresses the problem of Alzheimer’s disease (AD) diagnosis with Electroencephalography (EEG). The use of EEG as a tool for AD diagnosis has been widely studied by comparing EEG signals of AD patients only to those of healthy subjects. By contrast, we perform automated EEG diagnosis in a differential diagnosis context using a new database, acquired in clinical conditions, which contains EEG data of 169 patients: subjective cognitive impairment (SCI) patients, mild cognitive impairment (MCI) patients, possible Alzheimer’s disease (AD) patients, and patients with other pathologies. We show that two EEG features, namely epoch-based entropy (a measure of signal complexity) and bump modeling (a measure of synchrony) are sufficient for efficient discrimination between these groups. We studied the performance of our methodology for the automatic discrimination of possible AD patients from SCI patients and from patients with MCI or other pathologies. A classification accuracy of 91.6% (specificity = 100%, sensitivity = 87.8%) was obtained when discriminating SCI patients from possible AD patients and 81.8% to 88.8% accuracy was obtained for the 3-class classification of SCI, possible AD and other patients.

## Introduction

Dementia is a major public health issue worldwide. The impact on aging population grows at an alarming rate: the number of people living with dementia today is estimated at 46.8 millions, and expected to double by 2030 and triple by 2050 [1]. Alzheimer's disease is the most common form of neurodegenerative dementia, accounting for up to 75% of all dementia cases; it is a growing challenge to public health and the health care systems.

Alzheimer's disease (AD) is an irreversible neurodegenerative disease characterized by a decline in memory, language and other cognitive functions that affect a person’s ability to perform daily activities. The evolution of the disease frequently follows five stages. The “preclinical” stage is asymptomatic, but the brain lesions of Alzheimer's disease are present. In the subgroup of preclinical stage, the concept of subjective cognitive decline/impairment (SCI) has been proposed recently, defined by a self-experienced persistent decline in cognitive capacity in comparison with a previously normal status and with normal age-, gender- and education-adjusted performance on cognitive tests [2]. These subjective complaints are considered as a risk factor for AD [3]. In the second stage (“Mild Cognitive Impairment”–MCI), patients have some memory impairments, but maintain their functional capacities [4,5]; 6% to 25% of MCI patients later develop Alzheimer's dementia. Then, in the “Mild” stage of Alzheimer's dementia (MMSE>20), cognitive deficits are notable such as memory and learning impairments, which become more severe in the “Moderate AD” stage (MMSE between 10 and 20). In the final “severe” stage of the disease, almost all cognitive and motor functions are deeply deteriorated and patients are completely dependent on caregivers [6]. The average duration of survival of Alzheimer's disease patients is 5–8 years after clinical diagnosis [7,8]. Currently, no known medication exists for curing this pathology, but some therapeutic treatments at the early stage might delay the evolution of the disease [9,10]. Therefore, an early diagnosis of Alzheimer's disease in MCI and Mild AD stages becomes an important issue for the scientific and medical community.

Medical diagnosis of Alzheimer's disease is hard, particularly at the early stage of the disease, mainly because symptoms are often dismissed as normal consequences of ageing. In addition, other pathologies (e.g. dementia with Lewy bodies, fronto-temporal dementia, and vascular dementia) share some symptoms with Alzheimer's disease at the early stage. To diagnose Alzheimer's disease, extensive tests are required to eliminate all other possible causes. These tests include comprehensive neuropsychological evaluations, neurological examination, blood tests, brain imaging techniques and spinal fluid analysis if needed [11]. Diagnosis of Alzheimer's disease by non-invasive and inexpensive techniques will allow dispensing better care to patients.

In the last years, the potential use of electroencephalography (EEG) for diagnosing dementia pathologies, and in particular Alzheimer's disease has been extensively investigated [12–19]. EEG is a non-invasive, relatively inexpensive, and potentially mobile technology with high temporal resolution (on the order of milliseconds). It was mainly investigated as a tool for AD diagnosis, by comparing EEG recordings of AD patients only to those of control subjects (healthy subjects) [12,13,15,17,18].

It is widely admitted that Alzheimer's disease leads to a reduction in the complexity of EEG signals and changes in EEG synchrony. These modifications in EEG recordings have been used as discriminative features for AD diagnosis. Several methods were developed for assessing the complexity of EEG signals. The correlation dimension and the first positive Lyapunov exponent were frequently used [20–25]. It was found that EEG signals from AD patients exhibit lower values of such measures (lower complexity) than signals from age-matched control subjects. Other information-theoretic methods, entropy-based approaches in particular, have emerged as potentially useful EEG markers of Alzheimer's disease: epoch-based entropy [26,27], sample entropy [28], Tsallis entropy [29], approximate entropy [30,31], multi-scale entropy [32], and Lempel-Ziv complexity [33]. These methods link the complexity of a signal to its unpredictability: irregular signals are more complex than regular ones since they are more unpredictable.

Since Alzheimer's disease is hypothesized to induce functional disconnection between brain regions, other studies focused on detecting the changes in the synchrony between pairs of EEG signals. A large variety of measures has been developed to quantify EEG synchrony: correlation coefficient [34], coherence [34–36], Granger causality [34,37], phase synchrony [34,38,39], state space based synchrony [34,38,40], stochastic event synchrony [34,36,39,41,42] and mutual information [43]. All these studies reported decreased EEG synchrony in MCI and AD patients compared to healthy subjects.

To discriminate AD patients from healthy subjects, all the above-mentioned studies analyzed the EEG signals either in the time domain or in specific standard frequency bands: 0.1–4Hz (delta band), 4–8Hz (theta band), 8–12Hz (alpha band), 12–30Hz (beta band) and 30–100Hz (gamma band) [23,44], or in the whole frequency range between 4 and 30Hz [34]. Spectral analysis studies reported that Alzheimer's disease induces increased activity in the delta and theta frequency bands, as well as decreased activity in the alpha and beta bands [19,45–48]. Also, reduced spectral coherence between the two hemispheres was shown between alpha and beta frequency bands [14,49–52]. These spectral differences were also shown to be correlated with the severity of the disease [14,53,54]. Moreover, alpha rhythms are usually distributed in the occipital area for healthy subjects; in AD patients, they increasingly move towards anterior areas as the disease progresses [45,55,56]. Early stages of Alzheimer's disease have been associated with an increase of theta activity and/or a decrease of alpha activity. In more severe stages of Alzheimer's disease, an increase of both theta and delta activities has been observed together with a decrease of both alpha and beta activities, additionally to a reduction in the amplitude of the peak of alpha frequency band [57,58]. In all these studies, a 70%-85% correct detection rate is commonly achieved for different degrees of disease severity.

By contrast to all the above-mentioned investigations, the present study takes advantage of a database containing EEG data acquired from different patients in real clinical conditions. This database contains EEG data from patients with subjective cognitive impairment (SCI), possible AD patients (DSM IV definition), MCI patients and patients suffering from other pathologies, such as vascular dementia, psychosis, Lewy body dementia, and non neurodegenerative disorders (alcoholism, cerebral vascularitis, cerebellar abscess…). To the best of our knowledge, this is the first report of automatic discrimination, from EEG data, between SCI, MCI, and Mild to moderate possible AD patients. A similar work in the literature in terms of the exploited cohort is that of *Liedorp et al*. [59]. The authors used a large memory clinic database that contains EEG data of subjective memory complaints patients, MCI patients, AD patients, and patients with other dementias (psychiatric disorders, vascular dementia (VaD), fronto-temporal dementia, and Lewy bodies (DLB)). However, the authors investigated focal and diffuse abnormalities in different cognitive profiles of the database, based on a visual EEG assessment.

In the present work, both AD diagnosis and differential AD diagnosis are investigated. In the first case, possible AD patients are discriminated from SCI patients (AD diagnosis) only. In the second case, SCI patients and possible AD patients are discriminated from patients with MCI or other pathologies. The experiments involve the use of two features: an entropy-based complexity measure [26,27] and a synchrony measure [60,61], both computed in different frequency ranges and for different brain regions. The most relevant measures and the most relevant frequency range are selected with the Orthogonal Forward Regression (OFR) algorithm and the random probe method [62–64] to improve the accuracy of EEG classification using a Support Vector Machine classifier (SVM) [65,66].

## Material and methods

### Database description and pre-processing

The database was recorded in real clinical conditions between 2009 and 2013 at Charles-Foix Hospital (Ivry-sur-Seine, France). The EEG recordings were obtained at rest and with closed eyes using a Deltamed digital EEG acquisition system for a minimum of 20 minutes. Scalp electrodes were placed according to the modified International 10–20 system with 11 additional electrodes in a common reference montage using a sampling rate of 256 Hz. Thirty electrodes were placed on the scalp (Fp1, Fp2, F7, F3, Fz, F4, F8, FT7, FC3, FC7, FC4, FT8, T3, C3, Cz, C4, T4, TP7, CP3, CPz, CP4, TP8, T5, P3, Pz, P4, T6, O1, Oz, O2).

The patients who complained of memory impairment were referred to the outpatient memory clinic of the Charles-Foix Hospital where they underwent a battery of tests for brain disorders, including neuropsychological test, brain imaging and blood samplings. Patients with epilepsy were excluded. Each patient was given a diagnosis at the memory clinic on the basis of the clinical, brain imaging, psychometric findings, and discussions held by a multidisciplinary medical team, using the standard diagnostic criteria: DSM-IV, NINDS, Jessen criteria for SCI, Mc Keith criteria for Lewy body dementia [2,3,67]. We didn’t use EEG recordings to establish the diagnosis. This retrospective study was approved by the local ethical committee of the University Pierre and Marie Curie Paris 6. The database reflects what medical practitioners are facing in reality, as opposed to databases used in the literature [24,27,29,34–39] that are prone to experimental constraints that do not match the reality on the ground.

The database contains EEG data of 169 patients (mean age 75±11.2 years old, range 42–97 years old; 110 women). These patients are described in the Table 1.

**Table 1:** Clinical characteristics of the cohort. AD: Alzheimer’s disease; aMCI: amnestic MCI; oMCI: other MCI; SCI: subjective cognitive impairment; BZD: benzodiazepine.

	SCI (n = 22)	MCI (n = 58)		AD (n = 49)		Other pathologies (n = 40)			
	SCI (n = 22)	aMCI (n = 6)	oMCI (n = 52)	AD (n = 28)	Mixed (n = 21)	Lewy body dementia (n = 3)	Psychosis (n = 13)	Vascular dementia (n = 9)	Non neuro-degenerative disorders (n = 15)
Age Years old (mean ±SD)	68.9±10.3	74.5±12.7	75.2±10.8	80.8±10.5	81.6± 7.3	76.0±6.1	64.1±12.5	79.3±6.3	70.1±11.1
Gender : female N (%)	18 (81,8%)	3 (50%)	32 (61,5%)	19 (67,8%)	11 (52,4%)	3 (100%)	11 (84,6%)	4 (44,4%)	9 (60%)
Education year									
MMSE (mean ±SD)	28.3±1.6	28.2±1.2	24.5±4.9	18.3±6.1	17.9±7.0	16±5.6	23.8±3.3	21.9±5.2	18.4±7.0
BZD use N (%)	4 (18.2%)	1 (16.7%)	5 (9.6%)	8 (28.6%)	9 (42.8%)	0	4 (30.8%)	2 (22.2%)	7 (46.7%)
Antidepressant use N (%)	2 (9%)	1 (16.7%)	10 (19.2%)	12 (42.8%)	13 (61.9%)	1 (33.3%)	4 (30.8%)	2 (22.2%)	6 (40%)
Neuroleptic use N (%)	0	0	2 (3.8%)	5 (17.8%)	3 (14.3%)	1 (33.3%)	1 (7.7%)	1 (11.1%)	3 (20%)
hypnotic use N (%)	5 (22.7%)	1 (16.7%)	12 (23.1%)	7 (25%)	6 (28.6%)	0	2 (15.4%)	0	5 (23.8%)

For each subject, continuous epochs of 20 seconds, free from artifacts, were selected manually. They were then band-pass filtered with a third-order digital Butterworth filter between 1 and 30Hz.

### Methodology

The purpose of this study is to develop a method that consists in: (i) discriminating automatically possible AD patients from patients who came to the hospital with cognitive complaint but with normal age-, gender -and education-adjusted performance on cognitive tests i.e. SCI patients (AD diagnosis); (ii) discriminating automatically possible AD patients from SCI patients and patients with MCI or other pathologies (differential AD diagnosis).

For AD diagnosis experiments, only two groups were considered: the 22 SCI patients and the 49 possible AD patients. For differential AD diagnosis, two cases were investigated. In the first case, three groups were considered: the first group contained the 22 SCI patients (SCI patients); the second group contained the 49 AD patients (AD patients), and the third group contained all the remaining 98 patients with MCI or other pathologies (Other patients). In the second case of differential AD diagnosis, additionally to the three groups defined in the latter case, a fourth group was defined by separating the 58 MCI patients (MCI patients) from the 40 patients with other pathologies (Other pathologies). Note that the “Other patients” group in the first case and “Other pathologies” group in the second case are heterogeneous and contain patients with variable EEG signatures.

The proposed method exploits two EEG features: epoch-based entropy, which is a measure of signal complexity, and bump models, which quantify the EEG local synchrony. Both features are computed in different brain regions and in the four EEG frequency bands. The most relevant brain regions and frequency ranges are selected with the Orthogonal Forward Regression (OFR) algorithm [63,64] using a leave-one-subject-out cross-validation procedure and the random probe method [62]. The selected features are subsequently fed to a polynomial SVM classifier [65,66].

### EEG features

As mentioned above, epoch-based entropy and bump modeling were used for extracting relevant features from the EEG signal. These two measures have been reviewed earlier in [27,60]. A brief description is presented in the next two subsections.

#### Epoch-based entropy measure

Epoch-based entropy measure was introduced in [26,27] as a complexity measure for early screening of Alzheimer's disease. The reliability of this measure stems from the fact that it estimates the complexity of EEG signals not only locally over time (as classical complexity measures do), but also spatially by estimating the inter-channel complexity.

**Fig 1:**
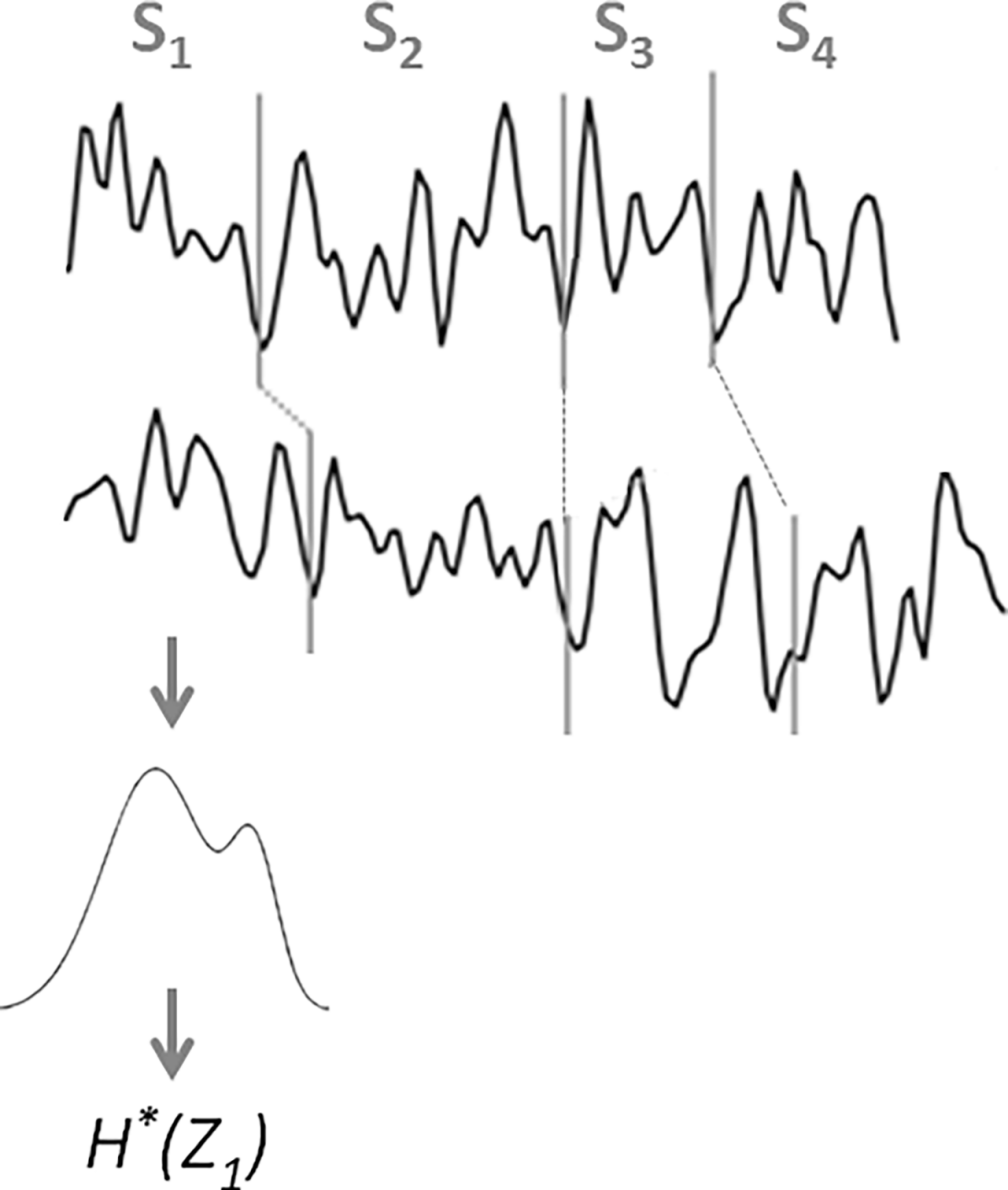
Illustration of multi-channel (*N* = 4, D = 2) EEG signal modeling with HMM.

The measure is computed on piecewise stationary epochs of EEG signal using a Hidden Markov Model (HMM) [68], which performs a local density estimation at the epoch level. As in our previous studies [27], EEG signals are modeled by a continuous left-to-right HMM (Fig 1). The states of the HMM correspond to the stationary parts of the EEG signal, and the transitions of the HMM correspond to the variations of the signal. The EEG signal recorded from a given subject is thus considered as a succession of epochs, obtained by segmenting the signal by the Viterbi algorithm [68] using the corresponding subject’s HMM. Thus, each obtained epoch corresponds to a state of the HMM and contains a given number of observations (sample points). For each epoch **S**_**i**_, the probability density function is modeled by a mixture of *M* Gaussian functions; each multivariate Gaussian has a diagonal covariance matrix (Fig 1).

Then each observation *z* in a given epoch **S**_**i**_ is considered as a realization *Z*_*i*_ of a random variable *Z* that follows a given observation probability distribution *P*_*i*_(*z*) modeled by the Gaussian mixture. Consequently, each stationary epoch of the signal is associated to a random variable, and the entropy H*(*Z*_*i*_) of the epoch **S**_**i**_ is that of an ensemble of realizations of *Z*_*i*_:

$$H^{*}(Z_{i})=-\sum_{z\in S_{i}}P_{i}(z).\log_{2}P_{i}(z)$$ (1)

By averaging the entropy over the *N* epochs of the EEG signal of the subject, an entropy-based complexity value *EpEn*(*Z*) of the signal, called “epoch-based entropy”, is obtained as:

$$EpEn(Z)=\frac{1}{N}\sum_{i=1}^{N}H^{*}(Z_{i})$$ (2)

To model the inter-relations between EEG time series recorded from *D* electrodes, an HMM is trained for each subject on a set of *D* EEG signals recorded from *D* electrodes. At time *t*, a hidden state emits a *D*-dimensional observation vector. By applying the Viterbi algorithm, each EEG signal is segmented into *N* epochs, and the entropy H*(*Z*_*i*_) of each epoch **S**_**i**_ is computed considering the probability density estimated by the HMM on the observations of the *D* epochs (Fig 1).

Although all *N* epochs are matched between EEG channels, the model does not constrain these epochs to be of equal length for all channels. Finally, by averaging the entropy over all the *N* epochs, an epoch-based entropy value associated to the multi-channel EEG of the subject is computed.

#### Bump models

Signal features can be extracted from time-frequency maps by means of sparse bump models [60]; those models consist of time-frequency patterns (“bumps”), lasting roughly 4 time periods centered at a specific frequency. The bump modeling approach allows capturing oscillatory events in EEG on a trial-by-trial basis, which in turn may be considered as reliable characteristic signatures in Local Field Potentials and EEG signals [61]. Those patterns are likely to be representative of transient local synchronization of neuronal assemblies, conveying key information on high-order cognitive and sensory processing.

Wavelet time-frequency maps are computed using complex Morlet wavelets. The (continuous) wavelet transform **W** of a time series **x** is obtained as:

$$W(k,s)≜\sum_{l}x(l)\Psi^{*}(\frac{l-k}{s})$$ (3)

where *Ψ*(*k*) is the mother' wavelet, *s* is a scaling factor, and * stands for complex conjugate. In this paper, we use the complex Morlet wavelet:

$$\Psi (k)=A.exp\left(\frac{-k^{2}}{2\sigma_{t}^{2}}\right).\exp\left(2i\pi f_{0}k\right)$$ (4)

where $\sigma_{t}^{2}$ and *f*_0_ jointly determine the number of oscillations in the wavelet. The complex Morlet wavelet family defined by 2*iπf*_0_*k* = 7 results in the optimal resolution in time and frequency; it has also proven to be suitable for EEG signals [58].

Oscillatory events (“bumps”) are extracted from the time-frequency maps using sparse bump modeling [69]. This procedure is described in more detail below.

Frequency-dependent z-score normalization [61,69] was applied to each trial:

$$z(f,t)=\frac{W(f,t)-\mu_{f}}{\sigma_{f}}$$ (5)

where *μ*_*f*_ and *σ*_*f*_ are the mean and the standard deviation respectively of the wavelet map **W**. The resulting z-score maps *z*(*f*,*t*) are approximated by bump models *z*_*bumps*_, which are sequences of basis functions *b* (“bumps”) with parameters *θ*_*k*_:

$$z(f,t)\approx z_{bumps}(\theta )=\sum_{k=1}^{N_{b}}b(\theta_{k})$$ (6)

with $\theta =(\theta_{1},\theta_{2},\ldots ,\theta_{N_{b}})$. This approximation retains the most salient oscillatory events in the z-scored map *z*(*f*,*t*). As pointed out earlier, we hypothesize that those events are characteristic for EEG dynamics, and are therefore relevant for diagnosing Alzheimer’s disease. In the present study, following [60,69], the basis functions *b*(.) were half ellipsoids, and the parameters *θ*_*k*_ were vectors of five parameters: position in time and frequency, width in time and frequency, and amplitude.

### Classification

#### Multi-class probabilistic SVM classifiers

This study involves a multi-class database containing four groups of patients: SCI patients, AD patients, MCI patients and patients with other pathologies. We are thus facing a *K*-class classification problem that was turned into a set of *K*(*K* − 1)/2 two-class problems [70].

To distinguish between each pair of classes, a polynomial SVM classifier with a margin calibration is used to overcome the issue of unbalanced datasets [71]. Therefore *K*(*K* − 1)/2 two-class SVM classifiers are trained in order to estimate pairwise posterior probabilities. The SVM outputs were mapped to posterior probabilities using Platt’s estimation method [72]. The global probability that an observation (a patient) described by the feature vector x, belongs to class *C*_*i*_ is computed as:

$$\mathrm{Pr}\left(C_{i}/x\right)=1/\left(\sum_{\begin{array}{c} j=1\\ j\neq i \end{array}}^{K}\frac{1}{{Pr}_{ij}}-(K-2)\right)$$ (7)

where *K* is the number of classes and *Pr*_*ij*_ is the probability of the observation belonging to the class *i*, estimated by the SVM classifier separating class *C*_*i*_ from class *C*_*j*_.

#### Feature selection

Epoch-based entropy and bump models features are computed on different frequency bands and on different brain regions. This leads to a large number of candidate input features to the SVM classifiers. Since the study also involves a multi-class problem, feature selection was performed to determine which features, among the candidate features, are the most relevant for discriminating each pair of classes. To rank the candidate features in order of decreasing relevance, we used the Orthogonal Forward Regression (OFR) algorithm [63,64] with a leave-one-subject-out cross-validation procedure, summarized as follows:

1. Select the candidate feature *f*_*i*_ that best correlates to the output to be modeled;
2. Project the output vector onto the null space of the selected feature. Orthogonalize the rest of features using Gram-Schmidt orthogonalization;
3. Remove the selected feature *f*_*i*_ from the list of candidate features;
4. Return to (1) until termination by the random probe method described below.

In order to select the features, we applied the random probe method [62]: 100 probes, i.e. random realizations of features, are generated, concatenated to the set of real data, and all features (real and probe) are ranked as described above. The user defines an acceptable risk that a feature might be kept although, given the available data, it might be less relevant than the probe. At each step of the selection procedure, the following steps are performed:

1. Obtain a candidate feature from OFR;
2. Estimate the value of the cumulative distribution function of the rank of the probe for the rank of the candidate feature. If the value is smaller than the acceptable risk, keep the feature and return to step 2 of OFR; otherwise, discard the considered feature and terminate the procedure.

## Experimental results

For all 169 subjects, epoch-based entropy and bump models were computed in different frequency bands and for different brain regions. On the basis of the results reported in the literature on Alzheimer’s disease detection with EEG, 16 features were considered as primary candidate variables: 7 features related to epoch-based entropy (*EpEn*) and 9 features related to bump models (BM), as reported in Table 2. The squared primary variables were considered as secondary variables in order to take into account possible non-linearities, so that the total number of candidate features was 32.

**Table 2:** The computed epoch-based entropy and bump model features for each subject.

Epoch-based entropy (*EpEn*)	Brain regions		
	All electrodes	Temporal (left and right)	Frontal+Occipital
Frequency range (Hz)	1–4 ; 4–8 ; 8–12 ; 12–30 ; 8–30	8–30	8–30
Bump models (*BM*)	Brain regions		
	Frontal	Occipital	Temporal (left and right)
Frequency range (Hz)	4–8 ; 8–12 ; 12–30	4–8 ; 8–12 ; 12–30	4–8 ; 8–12 ; 12–30

### AD diagnosis

AD diagnosis consists in discriminating SCI subjects (22 in the database) from AD subjects (49 in the database). As mentioned above, 32 candidate features were computed for each subject. Feature selection was performed using OFR algorithm with a leave-one-subject-out cross-validation procedure, as described in Section 2.4.2. Random variables (probes) were added to the feature set and only variables that ranked better than 90% of the probe were kept for classification. Therefore, the selected features are, in order of decreasing relevance:

a) *EpEn* computed on all electrodes, in the [8–12] Hz band,
b) *EpEn* computed on the Temporal region, in the [8–30] Hz band,
c) *BM* computed on the Frontal region, in the [4–8] Hz band,
d) *EpEn* computed on all electrodes, in the [8–30] Hz band,
e) *EpEn* computed on the Frontal + Occipital region, in the [8–30] Hz band.

This result shows that almost all selected features are related to the complexity measure, namely Epoch-based entropy (*EpEn*). Also, the optimal range on which EEG signal is the most informative for AD screening is 8 to 30Hz (alpha and beta bands).

**Fig 2:**
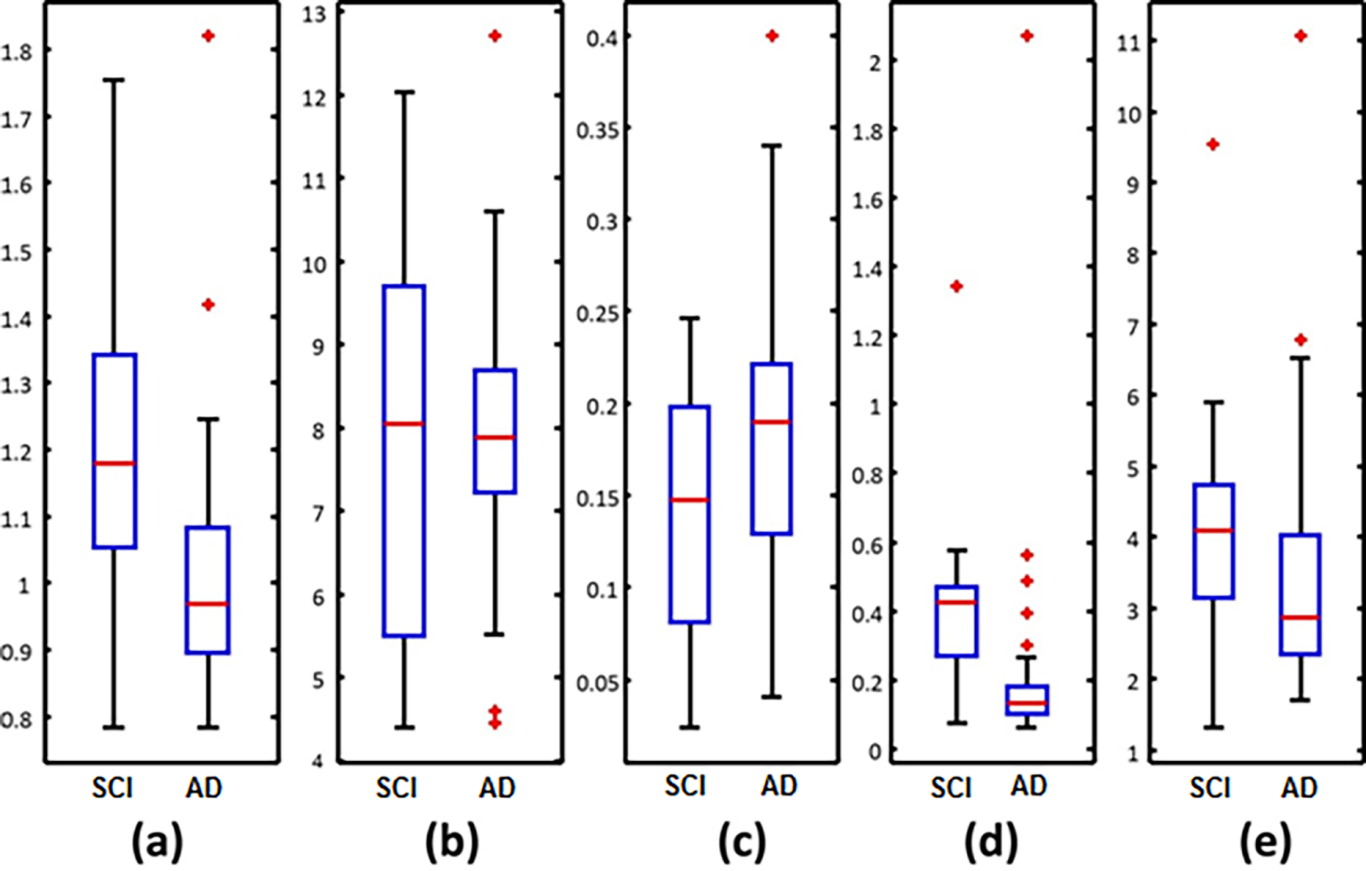
Box plots of the best features discriminating SCI patients from AD patients. The figures follow the ranking in order of decreasing relevance: (a) *EpEn* on all electrodes [8–12] Hz; (b) *EpEn* on temporal region [8–30] Hz; (c) BM on Frontal region [4–8] Hz; (d) *EpEn* on all electrodes [8–30] Hz; (e) *EpEn* on frontal + occipital region [8–30] Hz.

For further analysis, Fig 2 shows the box plots of features values obtained on SCI subjects and AD patients considering only the features that best discriminate these two groups. Fig 2A, Fig 2B, Fig 2D and Fig 2E show that AD patients have lower median values of epoch-based entropy than control subjects. This result is consistent with the literature: EEG signals from AD patients exhibit lower complexity values than age-matched control subjects in almost all channels [23,24,27,28]. In addition, Fig 2C indicates an increased EEG synchrony in the theta band for AD patients compared to SCI subjects. This result is consistent with previously published studies: AD induces an increased activity in the theta band [12,43,46]. It is interesting to point out that these results are still valid although the control subjects of this database are not healthy subjects since they have some memory complaints.

The selected features are subsequently used as inputs to a second-degree polynomial SVM classifier with soft margin. The performance of the SVM classifier was estimated by leave-one-subject-out cross-validation, which is known to provide an unbiased estimation of the generalization error [73]. Due to the small size of the database, the generalization error was not estimated on separate test data. Results of the classification showed that a correct classification rate of 91.6% is reached when discriminating SCI subjects from AD patients, with a specificity (proportion of well classified SCI patients) of 100% and a sensitivity (proportion of well classified AD patients) of 87.8%.

This result demonstrates the reliability of the used features and the proposed method for detecting Alzheimer’s disease. The result also shows a very good detection of SCI subjects with a specificity of 100% despite the fact that SCI subjects are not totally healthy subjects since they have some memory complaints.

### Differential AD diagnosis with three groups of patients

For differential AD diagnosis, the 169 subjects of the database were first organized into three groups: group 1 contains the 22 SCI subjects, group 2 contains the 49 AD patients, and group 3 contains the other 98 patients (with MCI or other pathologies).

Feature selection was performed on the 32 candidate features described in Table 2, in order to find the most relevant features for pairwise discrimination of the three groups. Table 3 shows the selected features for discriminating SCI from AD patients, SCI from “Other” patients, and AD from “Other” patients. The superscript indicates the order of the feature as ranked by OFR (“a” corresponds to rank 1, “b” to rank 2, etc).

**Table 3:** Best combination of features for discriminating SCI patients from AD patients (SCI vs. AD), SCI patients from those with MCI or other pathologies (SCI vs. Other), and AD patients from those with MCI or other pathologies (AD vs. Other).

Selected features	Epoch-based entropy			Bump Models	
	All electrodes	Temporal (left and right)	Frontal + Occipital	Frontal	Temporal (left and right)
SCI vs. AD	[8–12]^a^ [8–30]^d^	[8–30]^b^	[8–30]^e^	[4–8]^**c**^	-
SCI vs. Other	[8–12]^a^ [12–30]^c^	-	[8–30]^d^	[8–12]^**b**^	[8–12]^**e**^
AD vs. Other	[8–12]^f^ [12–30]^d^ [8–30]^b^	[8–30]^a^	[8–30]^c^	[12–30]^**g**^	[12–30]^**e**^

^a-g^ indicate the order of the feature as ranked by OFR (“a” corresponds to rank 1, “b” to rank 2, etc)

Table 3 shows that the majority of selected features are related to the complexity measure. Moreover, the optimal range on which EEG signal is more relevant for AD diagnosis is from 8 to 30Hz (alpha and beta bands).

A larger number of features is necessary for discriminating AD from “Other” patients than for discriminating SCI from AD and SCI from “Other” patients. This fact reflects the difficulty of detecting AD patients from patients with pathologies that share symptoms with AD.

**Fig 3:**
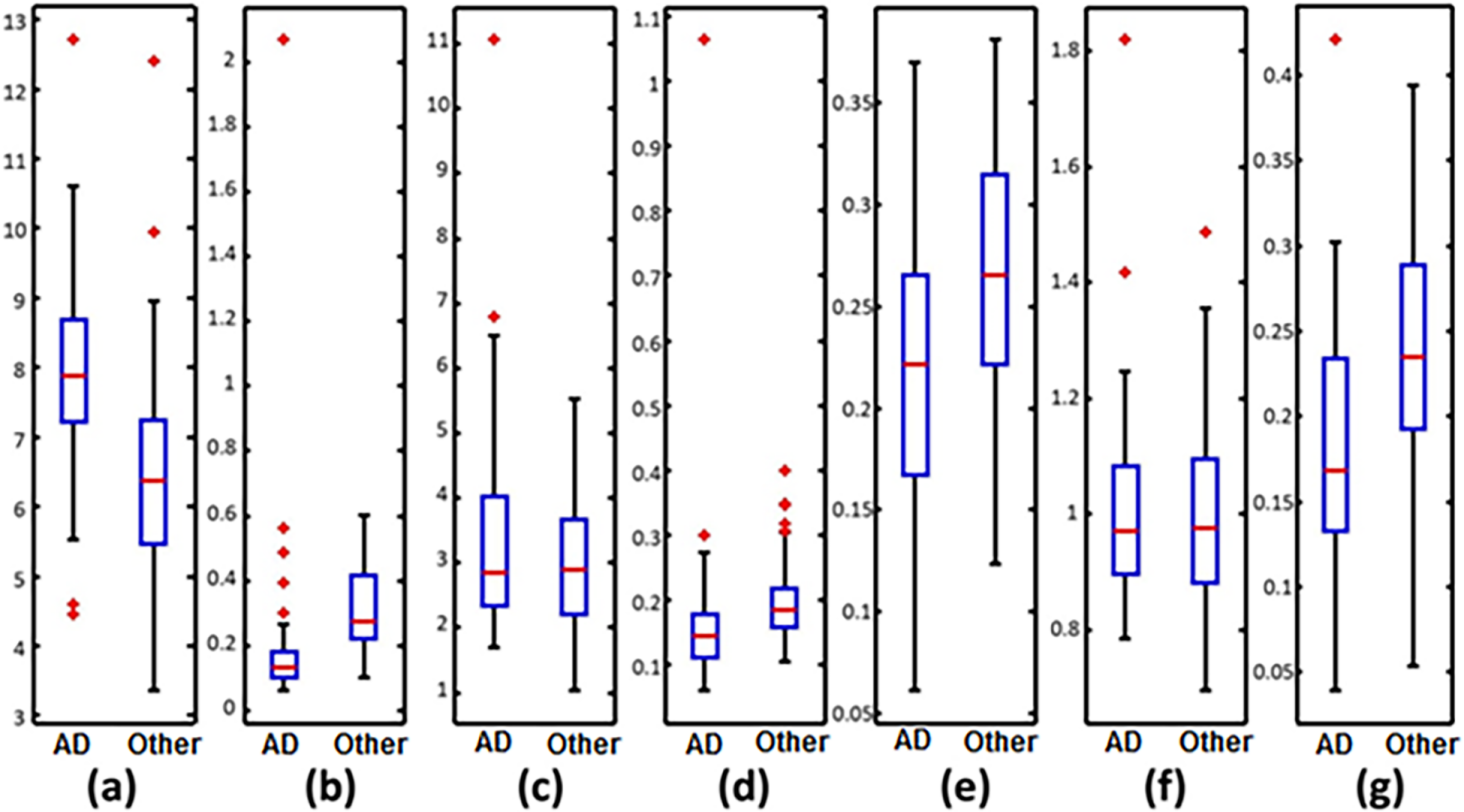
Box plots of the most relevant features for discriminating possible AD patients from “Other” patients (patients with MCI or other pathologies). Figures follow the same order given by the OFR algorithm as noted in Table 3: (a) *EpEn* on Temporal region [8–30] Hz; (b) *EpEn* on all electrodes [8–30] Hz; (c) *EpEn* on Frontal + Occipital region [8–30] Hz; (d) *EpEn* on all electrodes [12–30] Hz; (e) *BM* on Temporal region [12–30] Hz; (f) *EpEn* on all electrodes [8–12] Hz; (g) *BM* on Frontal region [12–30] Hz.

For further analysis, we present in Fig 3 the box plots of the 7 features values that best discriminate the 49 AD patients from the 98 patients with MCI or other pathologies.

Possible AD patients show a decreased EEG synchrony in the beta frequency range for both temporal (Fig 3E) and frontal (Fig 3G) regions. The Mann-Whitney test indicates that there is a significant difference (*p<0*.*001*) between the distribution of features of EEG signals of AD patients and the distribution of features of EEG signals of “Other” patients; this demonstrates the potential of the employed synchrony measure for detecting loss in EEG synchrony caused by Alzheimer’s disease.

In terms of complexity values, two behaviors appear in the alpha and beta ranges depending on the brain region:

(i) For EEG channels of all brain regions (Fig 3B and Fig 3D), it clearly appears that Alzheimer’s disease induces a reduction in complexity compared to the other pathologies. There is a significant difference (*p*<1e-6) between the distributions of features of the two populations;
(ii) For the temporal brain region (Fig 3A), EEG signals are more complex for AD patients than for the patients with MCI or other pathologies (*p*<1e-4).

The selected features are used as inputs of the corresponding three SVM classifiers associated to the three discriminations (SCI vs. AD, SCI vs. Other, AD vs. Other). The probabilistic outputs of the SVM classifiers are used to estimate the posterior probabilities of the classes following the procedure described in section 2.4.1. The classification performances estimated by leave-one-subject-out cross-validation are presented in Table 4.

**Table 4:** Confusion matrix for differential AD diagnosis with three groups of patients.

Three groups	SCI patients	AD patients	Other patients
SCI patients	**81.8%**	0%	18.2%
AD patients	8.2%	**87.8%**	4.1%
Other patients	6.1%	5.1%	**88.8%**

First, Table 4 shows that the proposed methodology leads to a high rate of correct classification of the subjects, higher than 82% (see the diagonal of the confusion matrix). Moreover, the detection rates are almost equivalent for the three groups (81.8% to 88.8%); this reflects the tradeoff made by our methodology between EEG specificity and EEG sensitivity for classifying the three groups.

Almost 82% of SCI patients are correctly detected, and no SCI patient (0%) was misclassified as AD patient. However, almost 18% of SCI patients were detected as patients with MCI or other pathologies. This result might be due to the fact that 59.2% of the 98 remaining patients of the database are MCI patients, which are very close to the SCI patients. This hypothesis is confirmed in section 3.3 below.

Table 4 also shows that 87.8% of AD patients are well detected. Among the remaining misclassified AD patients, two thirds are detected as SCI subjects (8.2%) and one third as suffering from MCI or other pathologies (4.1%). For the “Other” patients, 88.8% of them are well detected, and among the misclassified patients, half of them are considered as AD patients, the others as SCI patients.

For further analysis, we studied the effect of age on AD diagnosis as an etiological factor. To this end, we added age to the set of initial candidate variables and performed feature selection as described in Section 2.4.2. As a result, age was selected only for discrimination of SCI vs. AD, and not selected for SCI vs. Other and AD vs. Other, and the overall classification performance was found to decrease.

As a final test, the selected features for SCI vs. AD, without considering age in the set of initial candidates, were orthogonalized with respect to the vector of ages using Gram-Schmidt orthogonalization, thereby generating a new set of features that were decorrelated from age; these features were input to a new SVM classifier. The classification performance was found to be the same as obtained previously. This shows that the information on age present implicitly in the features had no influence on the classification results.

The results of the above two numerical experiments on age show that, for the differential diagnostics of interest in the present study, and given the available data, age is not a relevant factor.

### Differential AD diagnosis with four groups of patients

In the present subsection, the group including patients with MCI and other pathologies is split into two distinct groups: “MCI” group and “Other pathologies” group. The same procedure was used as in Section 3.2: pairwise classification between the groups was performed by 6 SVM classifiers, after feature selection. The performance was estimated by leave-one-subject-out cross-validation.

The results showed that for discriminating the 6 pairs of groups, EEG analysis should be carried out on the frequency range of 4–30 Hz. In contrast to the previous sections, where, in almost all cases, only the alpha and beta ranges were taken into account, the theta band is additionally considered. We also found that only one feature was selected for distinguishing MCI patients from the patients with other pathologies. This encourages the use of other features for better characterizing MCI patients.

The results of the classification of the four groups are shown in Table 5 in terms of confusion matrix. We first observe on the diagonal of the confusion matrix, that 82% of SCI patients and almost 90% of AD patients are well detected. However, we notice the difficulty in detecting MCI patients (60.4%) and patients with other pathologies (45%).

**Table 5:** Confusion matrix for differential AD diagnosis with four groups of patients.

Four groups	SCI	AD	MCI	Other pathologies
SCI	**81.8%**	0%	18.2%	0%
AD	6.1%	**89.8%**	2.0%	2.0%
MCI	5.2%	6.9%	**60.3%**	27.6%
Other pathologies	2.5%	5%	47.5%	**45%**

Moreover, the misclassified SCI patients are all classified as MCI patients. This result confirms our hypothesis stated in Section 3.2: SCI patients in the database suffer from memory complaints, which makes them similar to MCI patients based on our EEG descriptors. This result is particularly interesting in the framework of AD diagnosis, since this is the first EEG study, to the best of our knowledge, where SCI patients are discriminated from MCI and Mild AD patients: usually, MCI and Mild AD patients are discriminated from healthy subjects.

The results show that the present features are not very efficient for discriminating MCI patients from patients with other pathologies: only 60.3% of MCI patients are well detected, and among the misclassified MCI, 70% of them are considered as patients with other pathologies.

Since these four groups contain mixed patients with different impairments, we report in Table 6 the distribution of the misclassified patients in each group to give insight into the results.

**Table 6:** Distribution of the misclassified patients in the four groups. Refer to Table 1 that describes the cohort in details.

	SCI	MCI	AD	Other pathologies
Among the 49 patients of AD group: 5 are misclassified	2 AD 1 mixed AD	1 AD	/	1 mixed AD
Among the 58 patients of MCI group: 23 are misclassified	3 MCI	/	3 aMCI 1 MCI	2 aMCI 14 MCI
Among the 40 patients with Other pathologies: 22 are misclassified	1 vascular	5 vascular 9 non disorder 5 psychosis	2 Lewy	/

When analyzing the distribution of the misclassified patients in Table 6, we observe that:

a) the only AD patient associated to “Other pathologies” group has a mixed AD (AD with other pathology).
b) the 5 misclassified aMCI patients (among the 6 existing in the database) are associated to AD group and “Other pathologies” group. This result comforts the fact that amnestic form of MCI predicts the progression to neurodegenerative disease.
c) three misclassified MCI patients are associated to SCI group and two others are associated to AD group and “Other pathologies” group. Compared to amnestic MCI, the other forms of MCI are more confused with SCI subjects.
d) among the 21 misclassified patients from “Other pathologies” group, 19 patients are considered as MCI patients. The only patient from “Other pathologies” group considered as SCI subject has a vascular dementia. Also, two patients with Lewy body dementia (among the 3 existing in the database) are considered as AD patients. This result reflects that our selected features couldn’t differentiate between Alzheimer’s dementia and Lewy body dementia.

## Discussion and conclusion

In clinical practice, medical doctors have to discriminate patients suffering from Alzheimer’s disease from persons suffering from other types of dementia, or MCI, or from patients with subjective cognitive impairment. Alzheimer’s disease (AD) is consequently sometimes difficult to diagnose and discriminate from these pathologies, without using cerebrospinal fluid (CSF) biomarkers or single-photon emission computerized tomography (SPECT-scan).

Misdiagnosed patients suffer from unsuitable medical care, and have a societal cost. For instance, patients suffering from vascular dementia with prior AD diagnosis use substantially more medical services every year until their dementia diagnosis, resulting in incremental annual medical costs of approximately $9,500-$14,000 [74].

It is widely admitted that EEG is potentially very useful for AD diagnosis. Nevertheless, state-of-the-art publications have three limitations. First, most publications report studies conducted on small databases, of around 20 persons, containing EEG signals from only age-matched healthy subjects and from patients affected by Alzheimer’s disease. Second, the methods advocated for EEG-based AD diagnosis tend to have low specificity, hence poor detection of healthy subjects [58,75]. Third, for all these studies, the accuracy of AD diagnosis is not evaluated in a differential diagnosis context with respect to other pathologies.

The present study overcomes the above limitations, by analyzing a large database containing EEG data recorded in different pathologies, in real clinical conditions. In addition to AD and MCI patients, we considered patients with different pathologies, and SCI patients who joined the study with a suspicion of neurodegenerative disorder, but were diagnosed as not suffering from any objective cognitive deficit. To the best of our knowledge, no study so far has been carried out on AD diagnosis in a differential diagnosis context based on an automatic discrimination from EEG data.

In our framework, two tasks were performed in the present study: (i) discriminating AD patients from SCI patients (AD diagnosis); (ii) discriminating AD patients from patients affected by other pathologies (differential AD diagnosis). Based on measures of synchrony and complexity, we discriminated AD patients from SCI patients with high specificity, and discriminated AD patients from patients with other pathologies. The classifiers are Support Vector Machines, with feature selection by the random probe method; performance estimation is performed by leave-one-subject-out cross-validation.

We obtained a high accuracy for the classification of SCI vs. AD patients (91.6% accuracy, 100% specificity and 87.8% sensitivity). To the best of our knowledge, this is the first report of AD vs. SCI automatic classification based on EEG analysis. Knowing that the only reliable AD diagnosis is achieved by a post-mortem analysis of the brain [9], a reasonable goal was to reach an 85~90% accuracy. Recent scientific studies in the field of AD diagnosis reported high accuracy in classification tasks comparing AD patients and aged-matched healthy controls [12,13,15,17,18,24,27]. Our results are in the same order of accuracy as these studies–despite the fact that SCI patients, as opposed to control healthy subjects, may suffer from biological degradations [76,77].

In addition, by discriminating AD, SCI and Other patients including MCI, we showed that the classification accuracies remained similar for a three-group classification (81.8% to 88.8% accuracies). Thus our method provides a good tradeoff between specificity and sensitivity for the three groups. When analyzing the selected features for classification, our study reveals that Alzheimer’s disease induces a reduction of EEG complexity and an increase of EEG synchrony in the theta band, compared to SCI patients, considered in this work as control subjects. This is important, since it shows that the results of the literature on AD screening remain valid when comparing AD patients to SCI patients.

Finally, when splitting the “Other” group into “MCI” group and “Other pathologies” group (Section 3.3), all misclassified SCI patients were classified as MCI, which might be the consequence of the similarities between SCI patients and MCI patients: it has been shown that a proportion of SCI patients are actually at an early stage of MCI [78]. MCI patients were not correctly classified against patients with other pathologies. It is not very surprising that we failed to classify properly all these pathologies from the MCI stage, which could be caused by several different underlying mechanisms. Moreover, there would be a large overlap between the EEG signatures of these two groups due to causal heterogeneity in the “Other pathologies” group. A larger database would probably be necessary and other EEG features should be investigated.

In future work, we will focus the study of differential AD diagnosis on MCI patients in order to recover the best descriptors of this group of patients. We will also apply the methodology described in this paper on the other EEG data collected at Charles-Foix hospital to study the effectiveness of our method to discriminate, in blind manner, the different groups of patients.

## Abbreviations

AD: Alzheimer’s disease
BM: Bump models
EpEn: Epoch-based entropy
HMM: Hidden Markov Model
MCI: Mild Cognitive Impairment
aMCI: amnestic Mild Cognitive Impairment
MMSE: Mini Mental Status Examination
OFR: Orthogonal Forward Regression
Other: patients with MCI or other pathologies
SCI: subjective cognitive impairment
SVM: Support Vector Machine
